# Genome-wide association study for mammary structure in Canadian Angus cows

**DOI:** 10.1371/journal.pone.0237818

**Published:** 2020-08-27

**Authors:** Kajal Devani, Graham Plastow, Karin Orsel, Tiago S. Valente

**Affiliations:** 1 Department of Production Animal Health, Faculty of Veterinary Medicine, University of Calgary, Calgary, Alberta, Canada; 2 Department of Agricultural, Food and Nutritional Science, University of Alberta, Edmonton, Alberta, Canada; University of Florida, UNITED STATES

## Abstract

Functional and enduring mammary structure is pivotal for producer profitability, and animal health and welfare in beef production. Genetic evaluations for teat and udder score in Canadian Angus cattle have previously been developed. The aim of this study was to identify genomic regions associated with teat and udder structure in Canadian Angus cows thereby enhancing knowledge of the biological architecture of these traits. Thus, we performed a weighted single-step genome wide association study (WssGWAS) to identify candidate genes for teat and udder score in 1,582 Canadian Angus cows typed with the GeneSeek^®^ Genomic Profiler Bovine 130K SNP array. Genomically enhanced estimated breeding values (GEBVs) were converted to SNP marker effects using unequal variances for markers to calculate weights for each SNP over three iterations. At the genome wide level, we detected windows of 20 consecutive SNPs that explained more than 0.5% of the variance observed in these traits. A total of 35 and 28 windows were identified for teat and udder score, respectively, with two SNP windows in common for both traits. Using Ensembl, the SNP windows were used to search for candidate genes and quantitative trait loci (QTL). A total of 94 and 71 characterized genes were identified in the regions for teat and udder score, respectively. Of these, 7 genes were common for both traits. Gene network and enrichment analysis, using Ingenuity Pathway Analysis (IPA), signified key pathways unique to each trait. Genes of interest were associated with immune response and wound healing, adipose tissue development and morphology, and epithelial and vascular development and morphology. Genetic architecture from this GWAS confirms that teat and udder score are distinct, polygenic traits involving varying and complex biological pathways, and that genetic selection for improved teat and udder score is possible.

## Introduction

Mammary structure is a functional trait that has been associated with cow longevity [1, 2], cow health and welfare [3], as well as with maternal care and performance of calves [4–6]. Therefore, maintenance of functional mammary structure (teat size and shape, and udder suspension, size, and shape), plays an important economic role for cow-calf producers.

Previous studies have demonstrated that individual differences in bovine mammary structure is genetically influenced with heritability value estimates ranging from low to moderate [7, 8]. In addition, there is significant potential for identifying the genetic contributions and biological mechanisms that lead to individual variation observed in this complex system. To date, genome-wide association studies (GWAS) for teat and udder structure have focused primarily on dairy cattle and traits of economic importance to the dairy industry including attachment and size for both teat and udder, mastitis resistance, milking speed, and milk production [9–11]. Few GWAS have included *Bos taurus* beef breed populations. Vallee et al. [12] have performed GWAS for udder volume, teat size, and leg structure in Charolais cattle. In Fleckvieh cattle, Pausch et al. [13] identified QTL significantly associated with multiple mammary phenotypes such as udder depth, central ligament score, and teat length and thickness identified. And, in Angus-Nellore cross cows, Tolleson et al. [14] have performed GWAS for udder support score and teat size.

Since genetic evaluations and selection in beef cattle are evolving to include traits that impact efficiencies as well as animal health and welfare [15], the Canadian Angus Association (CAA) has recently developed a genetic evaluation for teat and udder score for Canadian Angus cattle. Covariance components and genetic parameters were estimated for teat size and udder suspension in Canadian Angus cows [7], however, the genes and molecular pathways associated with these traits remain unidentified. Therefore, the objective of this study was to perform a genome-wide association study aiming to identify genomic regions, potential candidate genes, and the biological mechanisms underlying teat and udder structure in Canadian Angus cattle.

## Materials and methods

All procedures involving cattle were reviewed and approved by the University of Calgary Animal Care Committee (Protocol AC16-0218). The collection of DNA samples for animal genotyping was done in accordance with the Canadian Code of Practice for the care and handling of farm animal [16]. Detailed methods and materials are described by Devani et al. [7].

### Phenotypes

Detailed descriptions of teat and udder scoring in Canadian Angus cows, population structure, and animal management were provided previously by Devani et al [7]. In short, teat and udder scores were recorded by 3 trained individuals for 1,735 Canadian Angus cows from 10 herds across Alberta. The Beef Improvement Federation (BIF) recommended scoring guidelines [17], that ranges from 1 (large bottle shaped teats and pendulous udders with compromised suspension) to 9 (the smallest teats and tightest udders), were used to assess cow teat and udder structure. In accordance with the guidelines all bred females within each herd were assessed, and the poorest mammary quarter was scored.

Cow pedigree information (4-generation, including 8,199 dams and 3,227 sires), cow parity (ranging from 1 to 16), date of birth and breed (Black or Red Angus), calf date of birth and sex were provided by the Canadian Angus Association (CAA). Contemporary groups (CG) were defined by herd, year, and season of calving. The CG with no variation in the observed traits or with less than 3 cows were excluded. Cows in later parities were grouped to manage the distribution bias within parities. Parity groups are defined as per Table 1.

**Table 1:** Parity groups and distribution of teat and udder scores within each parity group used for teat and udder score GWAS in Canadian Angus cows.

			Teat score			Udder score		
Parity group	Parities included	Number of cows	Min	Max	Mean	Min	Max	Mean
1	1	307	4	9	7.61	5	9	7.72
2	2	200	2	9	7.10	4	9	7.25
3	3–4	372	1	9	6.29	2	9	6.10
4	5–7	356	1	9	5.80	1	9	5.36
5	8–13	146	1	9	5.42	1	8	4.77

### Genotypes

Of the phenotyped cows, 1,582 were genotyped using the GeneSeek^®^ Genomic Profiler Bovine 130k BeadChip. The BeadChip contains 139,456 highly polymorphic SNPs that were selected for high minor allele frequency values and uniform genome coverage for *Bos taurus* cattle, with a mean distance of 19 kb between markers. Quality control was applied to exclude SNPs that had a call rate lower than 0.95, a minimum allele frequency lower than 0.01, monomorphic SNPs, or were deviant from Hardy-Weinberg equilibrium (χ^2^ >0.05). Animals were excluded for greater than 0.10 frequency missing genotypes or parent-progeny Mendelian conflicts [18]. After quality control, a total of 1,459 animals with genotypes comprised of 103,980 SNPs remained and were used for further GWAS analysis.

### Statistical analyses

Weighted single-step GBLUP (WssGBLUP) was used to estimate SNP effects as illustrated by Wang et al. [19]. The WssGBLUP was conducted using BLUPF90 family of software [18], wherein all phenotype, pedigree, and genomic information was combined simultaneously using Bayesian inference via Gibbs sampling. For both traits, the animal model included additive genetic effect and residual effect as random effects, contemporary group, breed, and parity group as fixed effects, and the number of days-between-calving-and-measure (linear effect) as covariate. Only for udder score, the number of days-between-calving-and-measure was included as covariate with quadratic effect. The general model can be represented as follow:

y=Xβ+Wa+e,

where ***y*** is the vector of phenotypic observations of teat or udder score, *β* is the vector of fixed effects, ***a*** is the vector of direct additive genetic effects, ***e*** is the vector of the random residuals effects, ***X*** and ***W*** are the incidence matrices of *β* and *a*, respectively. Assumptions included *a* ~ N (0, $H\sigma_{a}^{2}$), where ***H*** is the relationship coefficient matrix amongst animals and the genetic additive variance ($\sigma_{a}^{2}$). Also, *e* ~ N (0, $I\sigma_{a}^{2}$) where ***I*** is identity matrix and $\sigma_{e}^{2}$ is the residual variance. The *prior* distribution for genetic and residual variance components was an inverted Wishart and the posterior estimates were obtained using the POSTGIBBSF90 program.

In accordance with Aguilar et al. [20] the inverse of ***H*** matrix, combining both pedigree and genomic information was obtained as follow:

$$H^{-1}=A^{-1}+\left[\begin{array}{ccc} 0 & & 0\\ 0 & & G^{-1}-A_{22}^{-1} \end{array}\right],$$

where $A_{22}^{-1}$ is the inverse of numerator relationship matrix for all phenotyped and genotyped animals and ***G***^−1^ is the inverse of the genomic relationship matrix which was constructed in accordance to VanRaden [21], following:

G=ZDZ´q,

where ***Z*** is the SNP incidence matrix containing genotypes (0, 1 or 2) adjusted for allele frequency, ***D*** is a diagonal matrix with the inverse of expected SNP variance (initially ***D*** = ***I***), and *q* is a weighting factor for SNP variances. The weighting factor used was as in Vitezica et al. (2011), ensuring that the average diagonal in ***G*** is close to that of ***A***_22_. For each analysis, 700,000 iterations were generated, retaining every 50^th^ sample. The first 200,000 iterations were discarded as fixed burn-in. Data convergence was checked through graphical analysis of sampled values.

Estimates of SNP effects and weights for WssGWAS were obtained in accordance with the iterative process proposed by Wang et al. [19], as follows:

In the first iteration (*t* = 0): ***D*** = ***I***; ***G*** = ***ZDZ***´***λ***, where

$$\lambda =\frac{1}{\sum_{i=1}^{M}2p_{i}(1-p_{i})},$$

GEBV were calculated for the entire dataset using ssGBLUP,

GEBV were converted to estimates of SNP effect,

$$(U):{\hat{u}}_{\left(t\right)}=\lambda D_{\left(f\right)}Z´G_{\left(f\right)}ˉ¹{\hat{a}}_{g},$$

where ${\hat{a}}_{g}$ is the GEBV of animals that were genotyped,

The weight for each SNP to be used in the next iteration was calculated as:

$$d_{\text{i}(t+1)}=u^{2}{}_{\text{i}(f)}2p_{\text{i}}(1-p_{\text{i}}),$$

where i is the i-th SNP,

The SNP weights were normalized to keep the total genetic variance constant:

$$D_{\left(t+1\right)}=\frac{tr(D_{\left(0\right)})}{tr(D_{\left(t+1\right)})}D_{\left(t+1\right)},$$

*G*_(*t*+1)_ = *ZD*_(*t*+1)_*Z*´*λ* was calculated,

t = t + 1 and loop to step 2.

Three iterations of the above process were run, from step 2 to 7, in accordance with Wang et al [22], wherein both animal and SNP effects were updated and used to construct the weighted ***G*** matrix, update the GEBV, and to estimate the new SNP effects during the next iteration. The percentage of genetic variance explained by each SNP window (comprised of 20 consecutive SNPs) was also calculated in accordance with Wang et al [22] as:

$$\frac{Var(a_{i})}{\sigma_{a}^{2}}\times 100\%=\frac{Var(\sum_{j=1}^{10}Z_{j}{û}_{j})}{\sigma_{a}^{2}}\times 100\%,$$

where *a*_*i*_ is the genetic value of the *i*-th SNP window that consists of a region of 20 consecutive SNPs, $\sigma_{a}^{2}$ is the additive genetic variance, *Z*_*j*_ is the vector of gene content of the *j*-th SNP for all individuals, and ${û}_{j}$ is the marker effect of the *i*-th SNP within the *i*-th SNP window.

### Gene network analyses

The results from the WssGBLUP were used to identify genomic windows associated with teat and udder scores. Thus, the SNP windows that explained more than 0.5% of the total genetic variance for teat and udder score were selected for further exploration. For visual reference, a Manhattan plot was created, for each trait, using the R 3.6.2 package “ggplot2” [23]. In addition, chromosome number, start and end coordinates of each SNP window were used to identify candidate genes from the *Bos taurus* genome UMD3.1 assembly as the reference map through Ensembl Biomart Martview application (http://www.ensembl.org).

The identification of gene networks, pathways, and enrichment analysis (Gene Ontology and KEGG pathways) were performed using Ingenuity Pathway Analysis (IPA, Ingenuity Systems, Redwood City, CA. (http://www.ingenuity.com), which uses human gene ontology terms and KEGG pathways to develop networks. In addition, manual searches were performed to ascertain whether any of the genes had been associated with traits in bovine within previous studies.

SNP windows that explained more than 0.5% of total additive genetic variance were also used to identify QTLs already described. Using SNP window start and end positions the animal QTLdb database (release 40, access date: January 3, 2020) [24] was interrogated for previously associated QTL within these regions. Documented QTL were catalogued according to associations and causation.

## Results and discussion

### Phenotypes

Detailed description for phenotype collection are described previously [7]. Observed teat and udder scores, on 1,735 Canadian Angus cows that ranged in parity from 1 to 13, varied from score 1 (large bottle shaped teats and pendulous udders) to score 9 (small symmetrical teats and well suspended udders). To correct for distribution bias observed in higher parities (due to producer culling) parity groups were constructed (Table 1). In total, 1,582 Canadian Angus cows were genotyped to incorporate genomic information into genetic evaluations and to better understand the genetic architecture of teat and udder structure through genome-wide association studies (GWAS). The descriptive statistics for the data set is presented in Table 2.

**Table 2:** Descriptive statistics for teat and udder score in Canadian Angus cows for use in GWAS analyses.

Number of phenotyped cows	1,735
Number of animals in the relationship matrix	52,024
Number of sires	3,227
Number of dams	8,199
Number of herds	10
Number of contemporary groups	12
Number of genotyped cows after quality control	1,459
Number of SNPs after quality control	103,980

### Estimation of variance components

The variance components and heritability estimates are presented in Table 3 in which the heritability (SE) for teat and udder score were 0.35 (0.05) and 0.16 (0.04), respectively which is in line with previous estimates reported based on the phenotypes and pedigree, but not including genotypes for this population [7]. These values are also in accordance with previously reported estimations for beef [8, 12, 25], and dairy [9, 26] cattle. Moderate heritability estimates are indicative for potential genetic selection and to improve teat and udder structure in Canadian Angus cows.

**Table 3:** Estimates of mean posterior heritability (h^2^), number of SNP windows (and genes within) explaining more than 0.5% variance for teat and udder score in Canadian Angus cows.

	Teat Score	Udder Score
*Additive variance ($\sigma_{a}^{2}$)* (SE)	0.57 (0.09)	0.24 (0.05)
*Residual variance ($\sigma_{e}^{2}$)* (SE)	0.91 (0.07)	0.88 (0.06)
*h*^2^ (SE)	0.35 (0.05)	0.16 (0.04)
Number of SNP windows^a^ explaining > 0.5% variance	35	28
Total % variance explained	26.23	23.45
Number of genes identified in windows using Ensembl^b^	94	71
Number of genes identified in windows using IPA^c^	138	83

^a^SNP windows with 20 consecutive SNPs.

^b^Ensembl Genome Browser (http://www.ensembl.org/index.html) UMD3.1.

^c^Ingenuity Pathway Analysis (IPA), Redwood City, CA. (http://www.ingenuity.com).

### Genome wide association study

**Fig 1:**
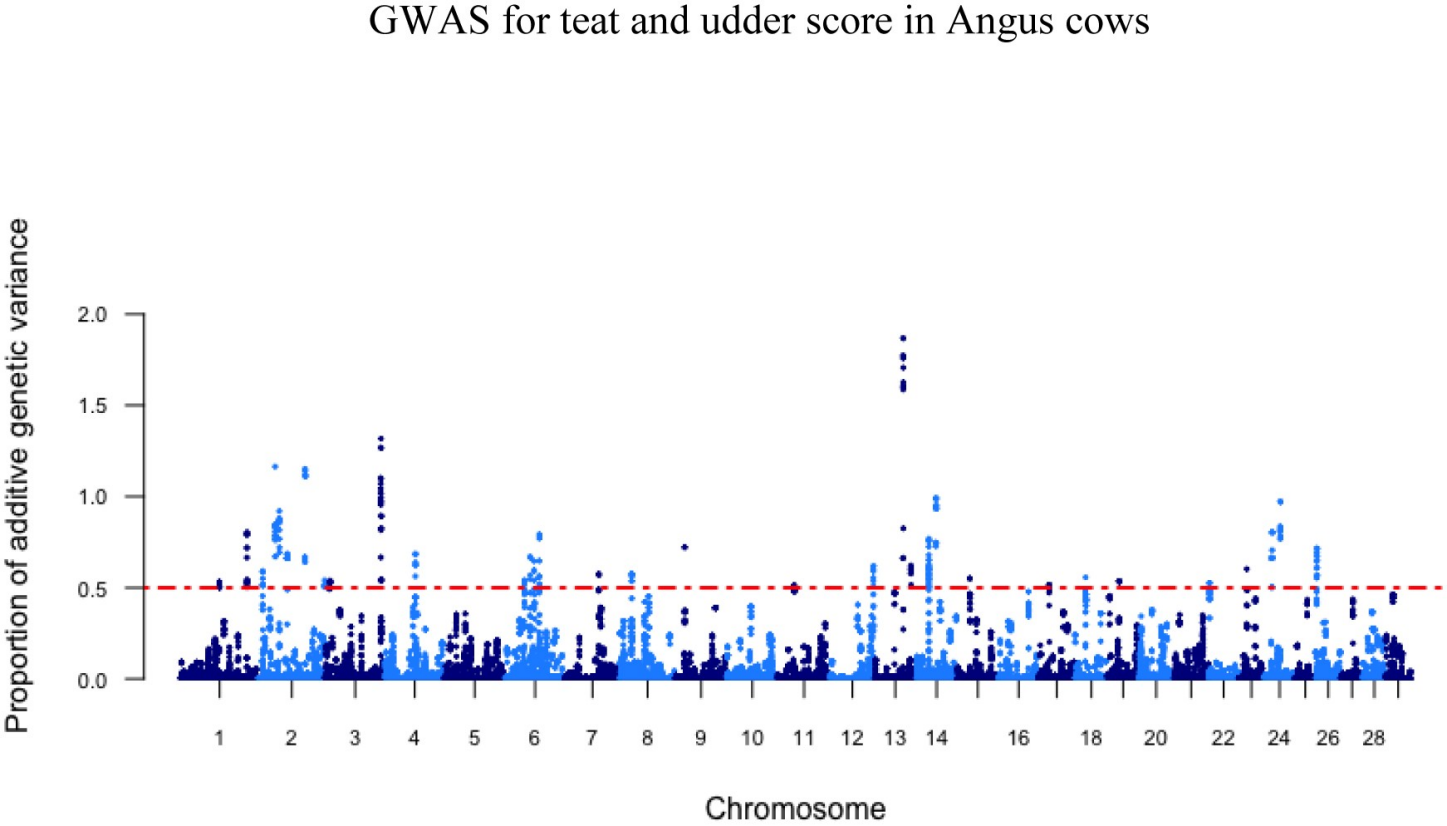
Manhattan plot depicting the percent variance explained by 20 consecutive SNP windows in teat score within Canadian Angus cows, dotted line indicative of 0.5% threshold for total additive genetic variance explained by SNP window.

**Fig 2:**
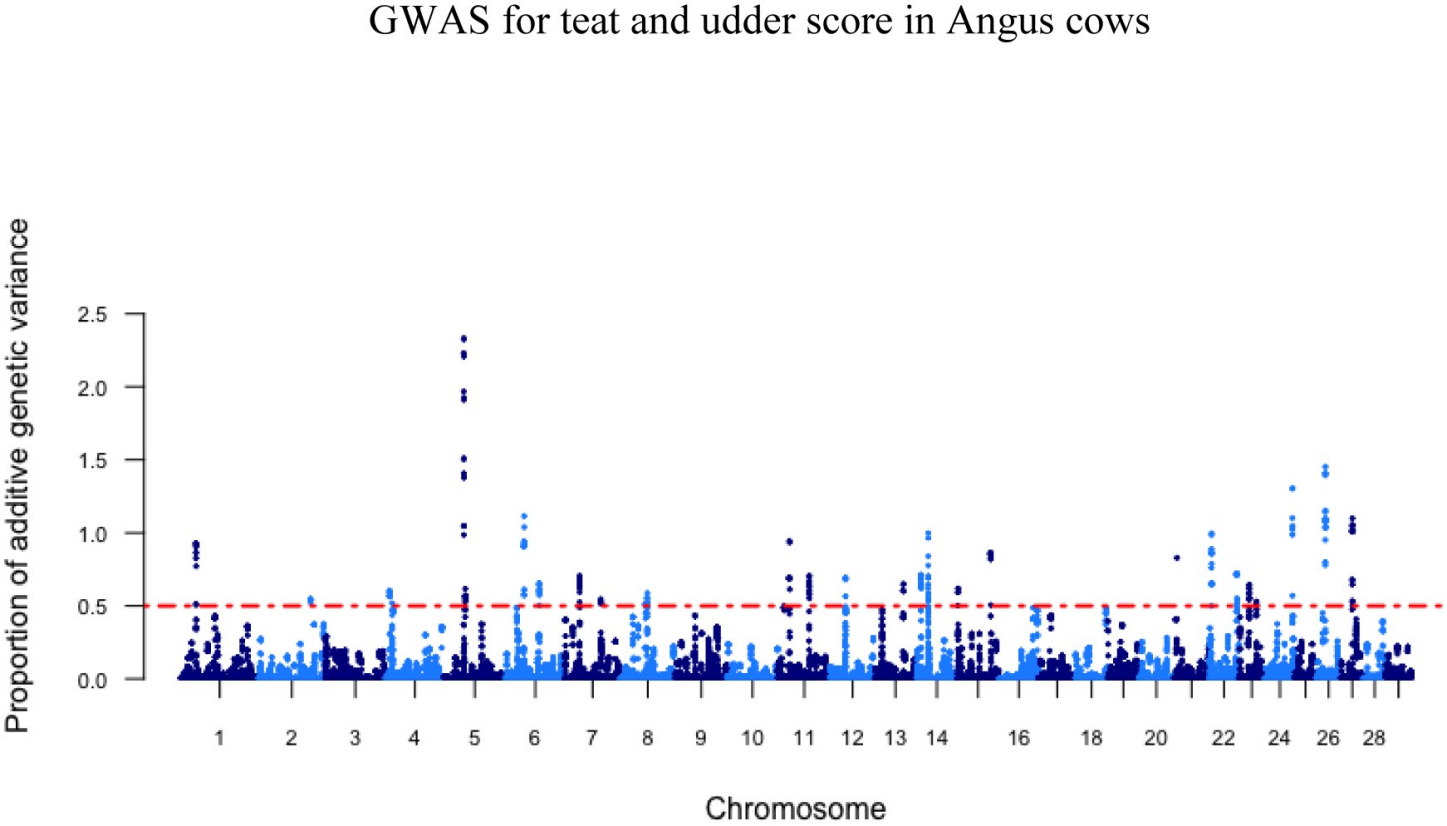
Manhattan plot depicting the percent variance explained by 20 consecutive SNP windows in udder score within Canadian Angus cows, dotted line indicative of 0.5% threshold for total additive genetic variance explained by SNP window.

The GWAS analyses for teat and udder score resulted in 35 and 28 associated windows of 20 consecutive SNPs, respectively, that explained more than 0.5% of the total additive genetic variance for these traits (Table 3). Of these windows, none explained more than 2.5% of the variation observed. In total, these SNP windows explained 26.23% and 23.45% of the total additive genetic variance for teat and udder structure in Canadian Angus cows, respectively. The SNP windows were distributed on *Bos taurus* autosomes and Manhattan plots are shown in Figs 1 and 2.

### Gene annotation

**Fig 3:**
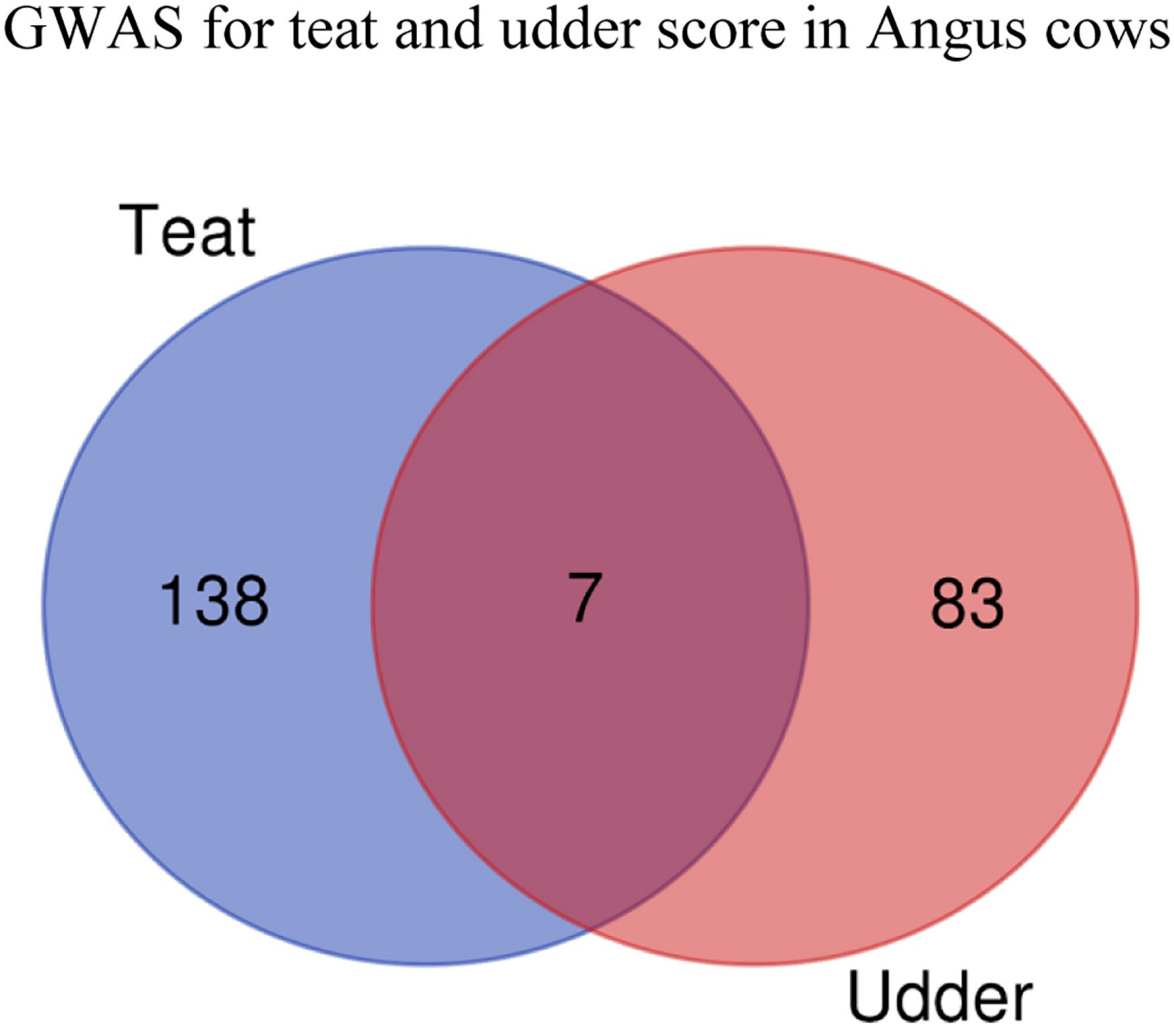
Venn diagram of number of genes identified in 20 consecutive SNP windows that explained more than 0.5% variance in teat and udder scores in Canadian Angus cows using Ingenuity Pathway Analysis software that references the human genome database.

Within these SNP windows, using the Biomart tool embedded in the Ensembl *Bos taurus* genes database version 94 (UMD3.1.), there were 94 and 71 annotated genes (including 11 open reading frames) identified (Tables 4 and 5). Only two SNP windows were in common for both traits in which 7 overlapping genes, 1 common microRNA, and 1 common open reading frame (*ANKRD60*, *APCDD1L*, *bta-mir-4449*, *C13H20orf85*, *RAB22A*, *RASL11B*, *RF00568*, *USP46*, *VAPB*) were identified. For teat score, most of the genes identified (18.09% and 13.83%) were on BTA2 and BTA6 respectively. For udder score, most of the genes identified (11.27% total) were on BTA2 and BTA22. Using IPA to extend this Ensemble list of genes identified, 138 and 83 characterized genes were identified for teat and udder score, respectively. From the two common SNP windows, 14 annotated genes were identified for both traits (Fig 3). These findings suggest that these are polygenic traits mainly influenced by distinct genes since there are many genomic regions with small additive effects on teat and udder structure, and that most of these are not commonly shared between the two traits. This is in line with previously reported genetic correlations between the traits ranging from 0.46 to 0.81 [7, 8, 27], as well as with other GWAS studies that found distinct genes associated with teat and udder score in beef [14] and dairy cattle [9].

**Table 4:** Summary of SNP windows that explained >0.5% of genetic variance for teat score, and the annotated gene list within each SNP window.

Chr^a^	SNP Window^b^		Var^b^	Annotated Genes
	Start, bp	End, bp		
1	135291628	135689292	0.80391	EPHB1
1	78724790	79274441	0.53317	TPRG1, LPP, BTA-MIR28
2	34266423	34625377	1.16462	IFIH1, FAP, GCG, SLC4A10
2	95189516	95683599	1.15161	ADAM23, DYTN, MDH1B, FASTKD2, CPO
2	42641470	43096191	0.91912	RPRM
2	58835964	59237220	0.68461	SPOPL
2	8624410	9040720	0.59139	CALCRL
2	135682198	135926465	0.54113	RCC2, BTA-MIR-2358, PADI6, PADI4, PADI3, PADI1
3	114252911	114711295	1.31744	TRPM8, SPP2, RF00494
3	114783422	115188884	0.89302	
3	10102567	10532687	0.5395	CRP, APCS, OR10J1, OR10J4, OR10J3
4	61897880	62506364	0.68588	RF00026, TBX20, DPY19L2, DPY19L1, RF00100
6	69917257	70291141*	0.79268	USP46, bta-mir-4449, RF00568, RASL11b, SCFD2
6	51119010	51469010	0.6677	RF00026
6	59266622	60027012	0.64628	KLF3, TLR10, TLR6, FAM114A1, TMEM156, KLHL5, WDR19
6	39701452	39762527	0.54138	
7	69168043	69641170	0.57407	SGCD
8	24326412	24683358	0.57493	SLC24A2
9	19040720	19547839	0.72312	LCA5, SH3BGRL2
11	33575714	34084906	0.51413	
12	88607685	88974025	0.61894	IRS2, RF00001, COL4A1
13	58632858	59072057*	1.86772	ANKRD60, C13H20orf85, PMEPA1
13	74283673	74765945	0.62068	SLPI, MATN4, RBPJL, SDC4, SYS1, TP53TG5
14	41390473	41836247	0.99112	RF00026
14	26923447	27198715	0.76933	TOX
14	26827140	26918229	0.72581	TOX
15	26193248	26660750	0.55046	CADM1
17	21923522	22452653	0.51443	RF00402
18	21396168	21954625	0.55713	CHD9, RBL2, AKTIP
19	24284098	24752222	0.53611	RAP1GAP2, OR1G1, RF00026
22	1728908	2137342	0.52597	SLC4A7, RF00001, EOMES
23	16883689	17283482	0.60259	ZNF318, ABCC10, DLK2, TJAP1, LRRC73, YIPF3, POLR1C, XPO5, POLH
24	33246227	33664170	0.97163	LAMA3, RF0026, ANKRD29, NCP1, RMC1, RIOK3, TMEM241
24	16358243	17028792	0.80323	
26	2648036	3271491	0.7161	ZWINT

^a^Chr = Chromosome number;

^b^Windows with 20 consecutive SNPs based on UMD3.1.;

^c^Var = percentage of additive genetic variance explained by each SNP windows.

**Table 5:** Summary of SNP windows that explained >0.5% of genetic variance for udder score, and annotated gene list within each SNP window.

Chr^a^	SNP Window^b^		Var^b^	Annotated Genes
	Start, bp	End, bp		
5	23377	23396	2.33234	CNTN1, RF00425
26	96869	96888	1.45364	PYROXD2, bta-mir-1287, HPS1, HPS1
24	94011	94030	1.30569	FECH, NARS, RF00026, ATP8B1
6	28850	28869	1.11534	
27	99137	99156	1.09971	TRMT9B, LONRF1, PRAG1
14	63172	63191	0.9982	SDR16C6, PENK
22	86990	87009	0.99806	GADL1
11	51699	51718	0.94444	
1	1222	1241	0.92687	RF00015, RF00026
15	68510	68529	0.86462	
21	84054	84073	0.82834	ASB7, LINS1, CERS3
22	89032	89051	0.72173	RAF1, MKRN2, MKRN2OS, TSEN2, PPARG, bta-mir-2373, SYN2
14	62411	62430	0.71244	
7	34146	34165	0.70732	
11	53244	53263	0.70389	RF00619
12	56289	56308	0.69346	MTUS2, SLC46A3, POMP
6	30554	30573	0.65273	USP46, bta-mir-4449, RF00568, RASL11B
13	60840	60859	0.64913	APCDD1L, VAPB, RAB22A, ANKRD60, C13H20orf85
23	90131	90150	0.64492	
15	65835	65854	0.62019	GPR83, MRE11, ANKRD49, AASDHPPT, KBTBD3, MSANTD4, GRIA4
5	23482	23501	0.61737	PTPRR, PTPRB, KCNMB4, CNOT2, RF00425
4	17370	17389	0.60515	
8	39962	39981	0.58718	TLE4
7	35820	35839	0.54559	ADRA1B, TTC1, RF00026
2	10673	10692	0.54502	TNS1, RUFY4, CXCR2, CXCR1, ARPC2, RF00406, GPBAR1, AAMP
23	90657	90676	0.53108	SOX4, CDKAL1
4	17597	17616	0.5181	DLX6, DLX5, SDHAF3
8	39816	39835	0.51698	PCSK5

^a^Chr = Chromosome number;

^b^Windows with 20 consecutive SNPs based on UMD3.1.;

^c^Var = percentage of additive genetic variance explained by each SNP windows.

### Gene network analyses

**Fig 4:**
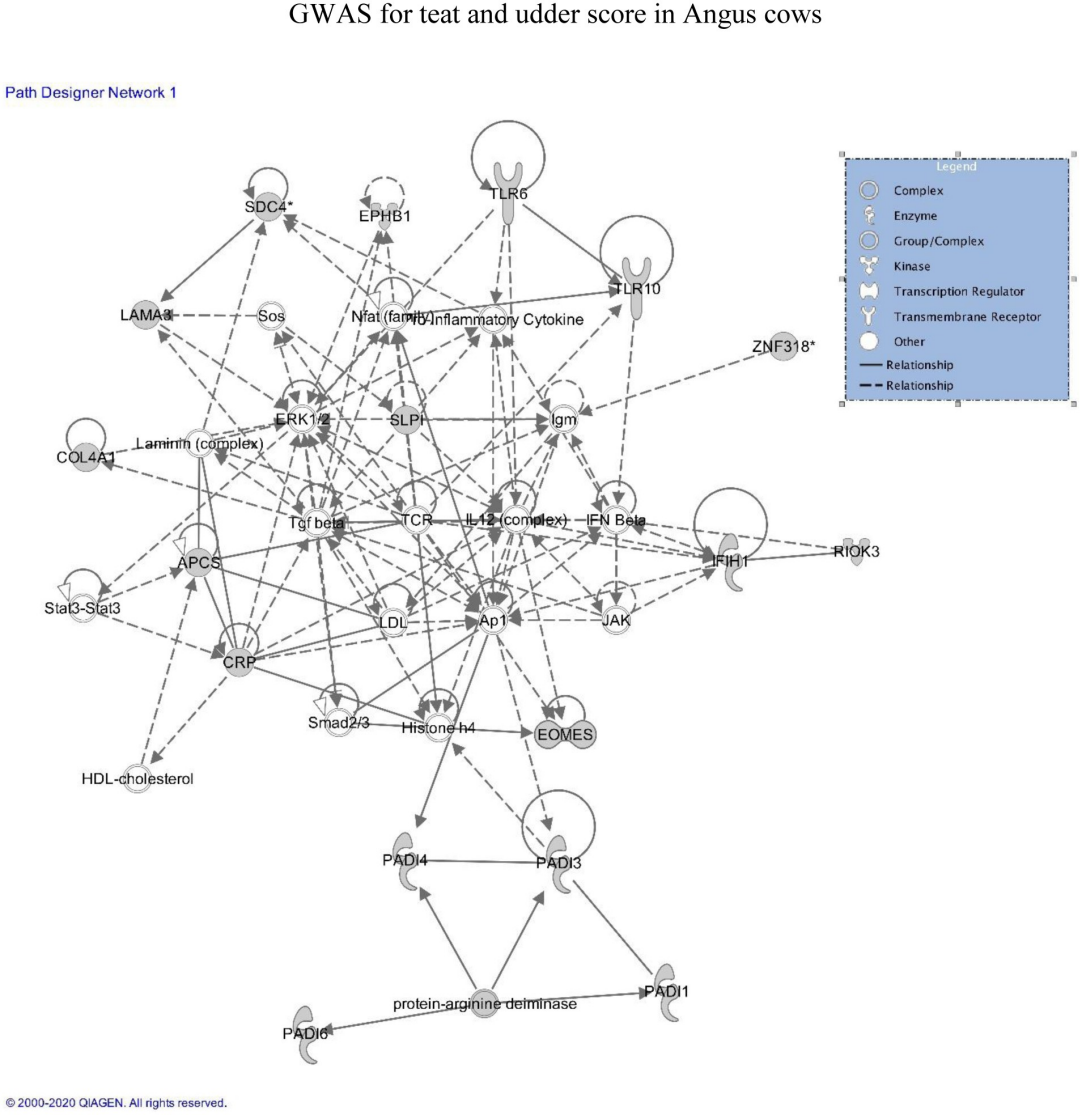
Genes identified in proximity to SNP windows that explained > 0.5% variance for teat score in Canadian Angus cows, and their involvement in the Cell Cycle, Cellular Assembly and Organization, and Cellular Function and Maintenance KEGG Pathway.

**Fig 5:**
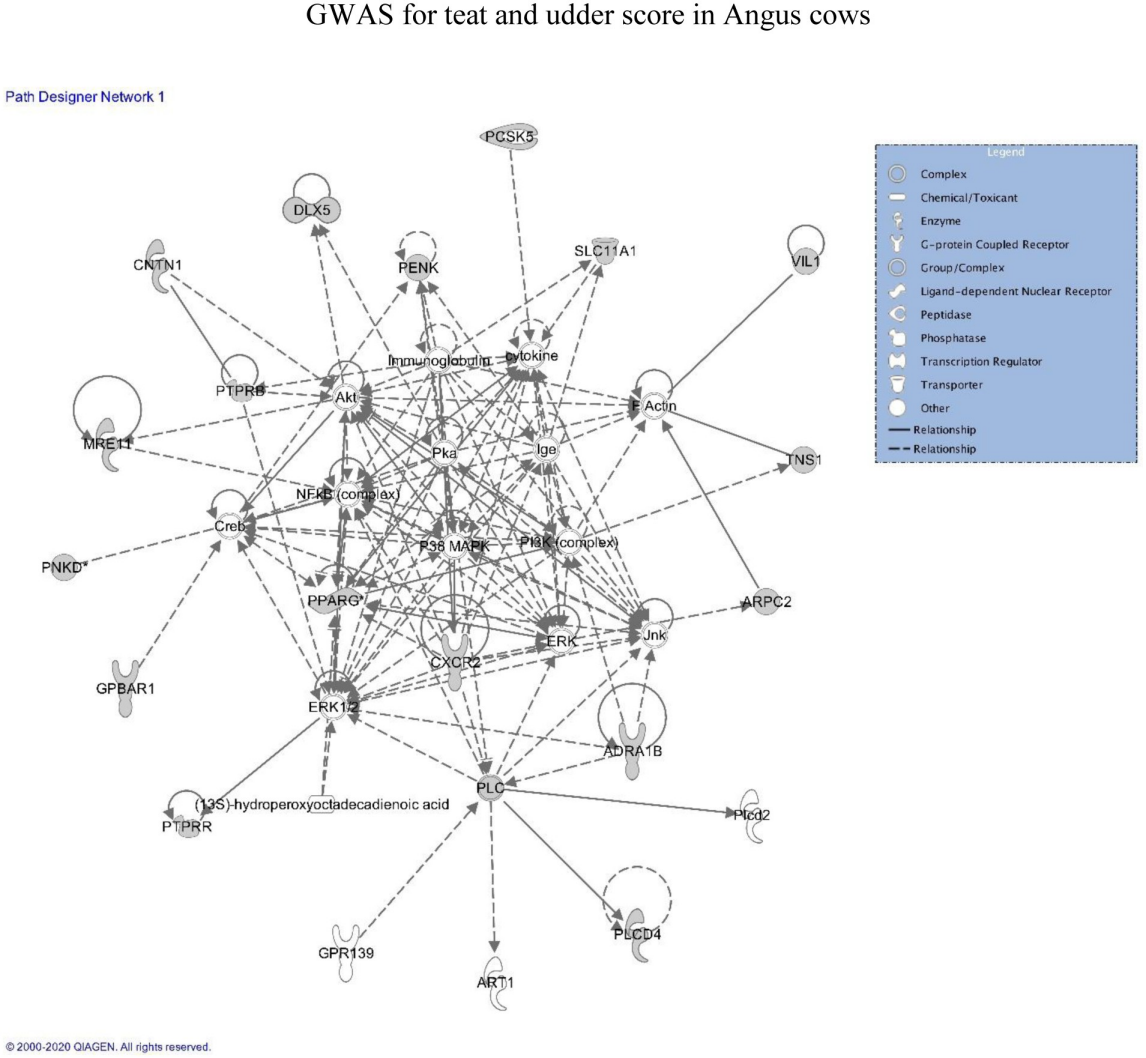
Genes identified in proximity to SNP windows that explained > 0.5% variance for udder score in Canadian Angus cows, and their involvement in the Cell-to-Cell Signaling and Interaction KEGG Pathway.

Gene enrichment analyses were performed by using the Ingenuity Pathway Analysis (IPA) software. The list of genes associated with each trait that was located within all SNP windows that explained more than 0.5% of the total additive genetic variance are presented in Tables 4 and 5 and were used as inputs for IPA. The most enriched gene networks identified associated (p < 0.05) with teat score are involved in *i* = Cell Cycle, Cellular Assembly and Organization, and Cellular Function and Maintenance (Fig 4); *ii* = Cardiovascular System Development and Function, Cell Morphology, and Organismal Development; *iii* = Cell-to-Cell Signaling and Interaction, Embryonic Development, and Reproductive System Development and Function; and *iv* = Gastrointestinal Disease, Gene Expression, and Organismal Injury and Abnormalities. The gene networks associated with udder score are involved in *i* = Cell-to-Cell Signaling and Interaction (Fig 5), Cellular Movement, and Connective Tissue Development and Function; *ii* = Cancer, Cell-to-Cell Signaling and Interaction, and Cellular Growth and Proliferation; *iii* = Hematological System Development and Function, Lymphoid Tissue Structure and Development, and Organ Morphology; and *iv* = Cell Signaling, Cellular Function and Maintenance, and DNA Replication, Recombination, and Repair.

In addition, in order to enhance understanding of the architecture of both traits, the input genes were grouped by associations with Diseases and Functions highlighted by IPA software (Table 6). The associations for both traits ranged from immune response and wound healing, lactation failure, fertility, udder health and morphology, and temperature sensitivity. Associations with cancer, specifically breast cancer, for a multitude of genes identified through this GWAS can be attributed to impaired gene function towards immune response, morphological integrity, and cell-cell binding. For teat score, genes were also grouped under functions pertaining to epithelial and vascular development, and thymus development, function, and associated diseases. On the other hand, for udder score, gene functions also included adipose tissue (development, quantity, function, and related diseases) and organ development. Of the genes identified within the 20 SNP windows that explained more than 0.5% of the total additive genetic variance for teat and udder score in Canadian Angus cows several are of particular interest in terms of knowledge and understanding of the trait architecture, such as *ADAM23* (on BTA2), *ANKRD60* (on BTA13), *CNTN1* (on BTA5), *PTPRR* (on BTA5), *USP46* (on BTA6), *and VEGFA* (on BTA23).

**Table 6:** Summary of diseases and functions associated with teat and udder score by using Ingenuity Pathway Analysis (IPA) software.

Function	Genes associated with teat score
Immune Response and Wound Healing	APCS, CADM1, CRP, EOMES, EPHB1, KLF3, LAMA3, RBL2, TLR6, TOX, VEGFA
Epithelial Cells, Vascular, Tubular Development	APCS, CALCRL, CRP, NPC1, SGCD, SLPI, TBX20, VEGFA
Thymus gland and functions	AKTIP, CADM1, COL4A1, EOMES, IRS2, RBL2SLC4A10, SLC4A7, TBX20, TOX, VEGFA
Fertility	ANKRD60, COL4A1, TOX
Udder health, morphology or index	C13H20orf85, ABCC10
Temperature sensitivity	TRPM8

A window of 20 consecutive SNPs on BTA2 explained 1.15% of the variance observed in teat score, this region contains the gene *ADAM23* (*ADAM Metallopeptidase Domain 23*) which encodes a disintegrin family of membrane anchored protein that has been associated with biological processes involving cell-cell interactions required for fertilization, muscle development and neurogenesis. Genes that play a role in cell-cell and cell-extracellular matrix are pivotal to tissue remodeling processes involved in structural development, wound healing, inflammation, and tumour cell invasion [28]. Aberrant expression and function of regulatory genes such as *ADAM23* can lead to loss of tissue architecture allowing higher proliferation of cancer cells. Verbisck et al. [29] demonstrated that cancer cells not expressing *ADAM23* had higher migration capacities. Thus, the gene has been associated with multiple types of cancer, including breast cancers [30, 31]. In addition, Elizondo et al. [32] have demonstrated *ADAM23* involvement in immune function has the expression of *ADAM23* in dendritic cells governs T cell proliferation and cytokine production. Integrity of mammary gland tissue architecture is essential to maintain both structure and function of the udder. In Holstein cattle, Fang et al. [33] found *ADAM23* to be significantly associated with milk protein production.

In addition, the *Vascular endothelial growth factor A* gene (*VEGFA*) which has been associated with milk protein and fat percentage in Holstein cows [34], was of particular interest for teat score. The *VEGFA* gene, which encodes a vascular endothelial growth factor is upregulated in cancer tumors and its expression is correlated with tumor stage and progression [35]. In bovine, the *VEGFA* gene has also been associated with angiogenesis and increased vascularity in ovarian follicles [36, 37], being considered an important regulator of placental development and function [38]. This gene plays an important function in the better supply of oxygen and nutrients to tissues, as well as in the tissue repair process after damage [39]. Healthy mammary glands need to be well supported with blood vessels, arteries and veins, these provide a continuous supply of nutrients to milk synthesising cells as well as regenerating cells. Thus, *VEGFA* is of particular interest towards understanding the biological architecture of mammary morphology. Conversely, in myocardial tissue *VEGFA* has been associated with vascular permeability and edema [40]. Udder edema has been linked increased incidence of mastitis and, in some instances, lower milk production [41]. It is also possible that udder edema may result in delayed calf suckling [42]. Teats that maintain size and structure under the duress of calf suckling are significant to the beef industry. Of interest, previous studies have alluded to eye-udder genetic correlations and consistent with this, *VEGFA* gene has been described to play a significant role in retinal epithelial cell development and proliferation [43].

Furthering the eye-udder link, a missense mutation in the *Receptor-type tyrosine-protein phosphatase R (PTPRR)* gene has been associated with high grade myopia [44, 45]. The *PTPRR* gene encodes a tyrosine phosphatase receptor type R protein known to be a signaling molecule that regulates cell growth, differentiation, and mitotic cycle. Altered protein tyrosine phosphatase signalling results in deregulated kinase activity oncogenic transformation [46]. This gene is implicated in numerous cancers [47], and specifically breast cancer due to its role in mammary epithelial cell biology [48]. Massive tissue remodeling occurs between parturitions in bovine mammary glands. Post lactational involution involves massive mammary epithelial cell apoptosis. In preparation for the next lactation, rapid proliferation of epithelial cells and cell differentiation to develop alveoli and ductal branches become predominant [49, 50]. Aoki et al. [51] suggest that (in mice, at least) protein tyrosine phosphatases are upregulated both during this proliferation phase of mammary gland and the involution phase, and down regulated during lactation. Regarding udder structure in bovine, *PTPRR* has been identified as upregulated or significantly associated with udder health and structure [52]. Herring et al. [14] found the *PTPRR* gene in close physical proximity with significant SNPs identified by GWAS for udder support and structure in Angus-Nellore crossbred cows. Engle et al. [53] found associations between *PTPRR* and stayability in the same cow population. These findings are in line with the observation that teat and udder structure contribute towards early culling in cows [1, 2, 54].

To this end, the *CNTN1* gene codes for Contactin 1, a glycosylphosphatidylinositol-anchored neuronal membrane protein and member of the immunoglobulin superfamily, that functions as a cell adhesion molecule. Contactin 1 is thought to play a role in the proliferation and differentiation on neurons [55]. It is noteworthy, that *CNTN1* has been associated with myopathy and muscular weakness in both humans and mice [56]. Compton et al [56] suggest nerve-muscle communication may be interrupted by the loss of glycosylate membrane binding in skeletal muscles within humans with *CNTN1* mutations. Maintenance of udder structure over repeated parities is dependent on muscular vigor. In addition, Shin et al. [57] used copy number variations to infer an association of *CNTN1* with milk production in Holstein cattle.

As mentioned previously, two SNP windows, and 7 annotated genes within them, overlapped between teat and udder score. One of the genes here was the *Ubiquitin Specific Peptidase 46* (*USP46*) gene. This is a protein coding gene which belongs to a large gene family of cysteine proteases that function as deubiquitinating enzymes (DUBs), and has been associated with maternal behavior in mice [58], mastitis resistance in sows [59], and breast cancer in humans [60]. DUBs may regulate gamma-Aminobutyric acid (GABA) action which is an inhibitory neurotransmitter but can also suppress inflammatory immune response while promoting regulatory immune response. GABA has been associated with human peripartum behaviour [61]. DUBs are also critical regulators of numerous ubiquitin dependent processes including synapse development and function. Ubiquitination of pre and postsynaptic proteins can regulate their stability, function and subcellular attachment [62]. Ubiquitination effects cellular processes by regulating the degradation or activation of proteins, this process is crucial for cell cycle progression, proliferation, and development. Previously discussed, profound proliferation and involution of both the extracellular matrix and mammary epithelial cells occur cyclically within the mammary gland. Thus, *USP46* may play a significant role in the maintenance of mammary health and structure. Relatedly, and also in common to both traits, was *Ankyrin Repeat Domain 60* (*ANKRD60*) which moderates protein-protein interactions between diverse families of proteins. Ankyrin repeats are tandemly repeated modules of approximately 33 amino acids that occur in a large number of functionally diverse proteins. Ankyrin repeat domain-containing protein 60 also consists of a ubiquitin-like-domain. Both the repeat motif and ubiquitin-like-domain are associated with cell process regulation and proteasomal degradation. Although the protein is not well characterized, in humans this gene is highly expressed in reproductive glands, and in bovine has also been associated with fertility [63]. From the gene enrichment activities within this study, IPA identified several genes that are associated with fertility and reproduction. Fertility is the most impactful variable on producer profitability, therefore, any associations between teat and udder score and fertility are important to explore further.

Previous GWAS for mammary structure traits in beef breeds identified that the *myostatin* gene (*GDF8*) was significantly associated with udder volume and teat size [12] in Charolais cattle. In addition, 8 genes (*SP5*, *GC*, *NPFFr2*, *CRIMI1*, *RXFP2*, *TBX5*, *RBM19* and *ADAM12*) that are in close proximity to 7 QTLs significantly associated with udder depth, central ligament score, and teat length and thickness were identified in Fleckvieh cattle [13]; and in Angus-Nellore cross cows, Tolleson et al. [14] found 3 SNPs associated with udder support score and teat size that are in close proximity to the *vitamin D receptor* gene (*VDR*), and *interleukin 22* gene (*IL22*). It was within this study that Tolleson et al. [14] also reported the association of the *protein tyrosine phosphate receptor type R* gene (*PTPRR*) with teat and udder structure. Enrichment analysis of previously identified associations with teat and udder structure includes genes related to immune response, cell signaling, tissue remodeling and organ development, supporting GWAS findings from this study.

### QTL database

To increase understanding about the molecular architecture of teat and udder structure in Canadian Angus cows, SNP windows from this GWAS were aligned to the publicly available Cattle Quantitative Trait Locus (QTL) Database (Cattle QTLdb). Documented QTLs were catalogued according to associations and causation, summarized in Table 7. The highest proportion of QTL identified were associated in previous studies with milk production (31%), reproductive production (16%) and body conformation (15%). Possibly a reflection of the amount of studies on teat and udder structure, particularly in beef cattle, only 2% and 7% of QTL previously associated within these windows were associated to teat and udder structure, respectively. The presence of QTLs within the windows identified from this study with those previously associated with mastitis resistance and immune function supports their important role in these traits.

**Table 7:** Previously identified QTLs and associated functions 344 within the windows of 20 consecutive SNP markers that explained more than 0.5% variance in teat and udder score in Canadian Angus cows.

	TEAT		UDDER	
	Associations / QTL^a^	Proportion	Associations / QTL^a^	Proportion
Body Conformation	395	15.38%	164	18.43%
Fat Desposition	102	3.97%	71	7.98%
Feed Efficiency	42	1.63%	36	4.04%
Growth	220	8.56%	105	11.80%
Immunological Function	82	3.19%	67	7.53%
Longevity	94	3.66%	4	0.45%
Mastitis	103	4.01%	21	2.36%
Milk Production	800	31.14%	275	30.90%
Reproductive Performance	412	16.04%	82	9.22%
Sensitivity to temperature	9	0.35%	2	0.22%
Social behaviour	4	0.16%	1	0.11%
Teat Conformation	50	1.95%	12	1.35%
Udder Conformation	178	6.93%	16	1.80%

^a^Number of associated QTLs.

## Conclusion

The objective of this study was to incorporate genomic information into the genetic evaluation for teat and udder score in Canadian Angus cattle in order to enhance knowledge and understanding of the genetic architecture of the traits. Using WssGWAS, multiple SNP windows were identified to explain 0.5% or more of the variance observed in teat and udder score for Canadian Angus cows, none surpassing 2.33%, implying that the traits are polygenic. Using IPA software for gene enrichment analysis we found genes associated with cancer, immune response and temperature sensitivity. Only 2 such SNP windows were common to both traits implying that the genetic architecture and biological pathways involved are distinct for the traits, and associated with epithelial cell, vascular, and tubular development, and thymus gland functions for teat score, and adipose tissue development, organ development and vigour, lactation failure and fertility for udder score. Previously described associations with QTLs within SNP windows of significance for teat and udder score in Canadian Angus cows also provided information on the biology behind teat and udder score within this population. Persisting good teat and udder structure supports producer profitability and animal health and welfare. This study therefore fosters an appreciation of the complexity of the traits and the need for accurate and appropriate genetic selection tools for teat and udder in beef cattle.
